# Unusually persistent Gα_i_-signaling of the neuropeptide Y_2_ receptor depletes cellular G_i/o_ pools and leads to a G_i_-refractory state

**DOI:** 10.1186/s12964-020-00537-6

**Published:** 2020-03-30

**Authors:** Isabelle Ziffert, Anette Kaiser, Stefanie Babilon, Karin Mörl, Annette G. Beck-Sickinger

**Affiliations:** Institute of Biochemistry, Faculty of Life Sciences, Leipzig University, Brüderstr. 34, D-04103 Leipzig, Germany

**Keywords:** Neuropeptide Y, Neuropeptide Y receptors, GPCR, Desensitization, Internalization, G protein

## Abstract

**Background:**

A sensitive balance between receptor activation and desensitization is crucial for cellular homeostasis. Like many other GPCR, the human neuropeptide Y_2_ receptor (hY_2_R) undergoes ligand dependent activation and internalization into intracellular compartments, followed by recycling to the plasma membrane. This receptor is involved in the pathophysiology of distinct diseases e.g. epilepsy and cancer progression and conveys anorexigenic signals which makes it an interesting and promising anti-obesity target. However, Y_2_R desensitization was observed after daily treatment with a selective PYY_13–36_ analog in vivo by a yet unknown mechanism.

**Materials:**

We studied the desensitization and activatability of recycled Y_2_R in transiently transfected HEK293 cells as well as in endogenously Y_2_R expressing SH-SY5Y and SMS-KAN cells. Results were evaluated by one-way ANOVA and Tukey post test.

**Results:**

We observed strong desensitization of the Y_2_R in a second round of stimulation despite its reappearance at the membrane. Already the first activation of the Y_2_R leads to depletion of the functional cellular Gα_i/o_ protein pool and consequently desensitizes the linked signal transduction pathways, independent of receptor internalization. This desensitization also extends to other Gα_i/o_-coupled GPCR and can be detected in transfected HEK293 as well as in SH-SY5Y and SMS-KAN cell lines, both expressing the Y_2_R endogenously. By overexpression of chimeric Gα_qi_ proteins in a model system, activation has been rescued, which identifies a critical role of the G protein status for cellular signaling. Furthermore, Y_2_R displays strong allosteric coupling to inhibitory G proteins in radioligand binding assays, and loses 10-fold affinity in the G protein-depleted state observed after activation, which can be largely abrogated by overexpression of the Gα_i_-subunit.

**Conclusion:**

The unusually persistent Gα_i_-signaling of the Y_2_R leads to a state of cellular desensitization of the inhibitory Gα_i_-pathway. The strong allosteric effects of the Y_2_R-Gα_i_-interaction might be a mechanism that contributes to the burst of Gα_i_-signaling, but also serves as a mechanism to limit the Y_2_-mediated signaling after recycling. Thus, the cell is left in a refractory state, preventing further Gα_i_-signaling of the Y_2_R itself but also other Gα_i/o_-coupled receptors by simply controlling the repertoire of downstream effectors.

**Video abstract**

**Graphical abstract:**

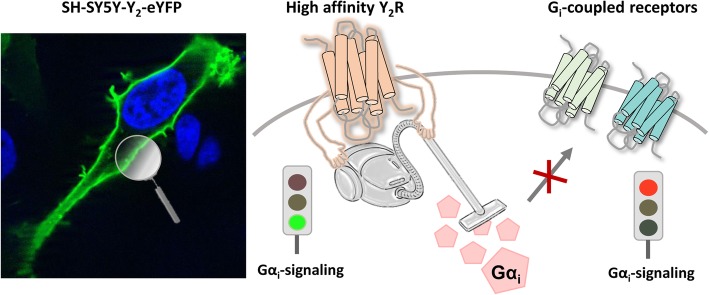

## Background

G protein-coupled receptors represent a major family of cell surface receptors with approximately 800 different subtypes that share a common architecture of seven transmembrane helices connected by three intra- and extracellular loops. Members of the GPCR transduce a large spectrum of extracellular signals and consequently different regulatory mechanisms are fundamental to protect cells against overstimulation. One of the major processes limiting GPCR signaling is the reduction of receptor sensitivity towards a particular stimulus over time. This mechanism, defined as desensitization, includes a complex series of events e.g. receptor phosphorylation, arrestin-mediated internalization, receptor recycling, lysosomal degradation and decrease in mRNA levels [1–3]. However, the regulatory mechanisms beyond the sensitive equilibrium of receptor activation and desensitization are complex and likely distinct for individual receptors. These mechanisms are of great interest since many pharmacological agents targeting GPCR display diminished effectiveness over time [4–7]. Moreover, such knowledge is required for applications that use GPCR as a shuttle system for intracellular drug delivery [8, 9]. To date, approximately 35% of approved drugs address GPCR and the number of promising targets increases steadily [10]. This includes the neuropeptide Y hormone receptor family, consisting of four receptor subtypes – Y_1_R, Y_2_R, Y_4_R, and Y_5_R. Activated by the three endogenous ligands neuropeptide Y (NPY), peptide YY (PYY) and pancreatic polypeptide (PP), the Y receptors form a multi-ligand/multi-receptor system and contribute to a large variety of physiological processes within the human body. Beside the regulation of food intake [11] and gastro-intestinal secretion, they also control blood pressure [12] and are involved in the pathophysiology of cancer progression as well as mood disorders [13, 14]. The Y_2_R, which is predominantly expressed in neuronal but also in peripheral tissue like liver, blood vessels and spleen, plays a central role in the development of new therapeutic drugs. Regarding its anorexigenic properties and its overexpression on specific tumor subtypes (glioblastoma; neuroblastoma) the Y_2_R represents a promising target for the treatment of obesity as well as for selective addressing of malignant tissue [15, 16]. However, intense Y_2_R desensitization was identified in mice and rats after daily treatment with a selective metabolically stable PYY_13–36_ analog [17]. This study indicates the importance of understanding the underlying mechanism that controls and regulates receptor activity to improve the effect of administrated drugs. Previous investigations based on Y_2_R endocytosis and intracellular trafficking demonstrated an arrestin-dependent internalization, subsequent endosomal sorting and transport back to the cell membrane, which is highly regulated through distinct motifs within the C-terminus [18]. Moreover, *Wanka* et al. revealed different binding modes of arrestin 3 (arr-3) at the human Y_1_R and Y_2_R [19]. In consequence of the “tail” conformation, Y_1_R binds Gα_0_-protein as well as arr-3 simultaneously forming a supercomplex. In contrast, no supercomplex formation was observed for the Y_2_R. Owing to the “core” conformation, binding of arrestin to Y_2_R results in the dissociation of G protein, thereby terminating both the binding of the G protein to the receptor as well as the G protein-mediated signaling. Based on these findings we investigated the desensitization process of the human neuropeptide Y_2_R and identified a novel mechanism for signal suppression. We demonstrate here that activation of the Y_2_R results in an unusually persistent Gα_i_-mediated signaling, which is facilitated by strong allosteric coupling of the receptor to inhibitory G proteins and is terminated by the depletion of the functional cellular G protein pool. This leads to a state of cellular desensitization of the inhibitory Gα-pathway for both the recovered Y_2_R as well as other Gα_i_-coupled receptors, protecting the cells against overstimulation by limiting the strong Y_2_R-mediated inhibitory G protein signaling.

## Methods

### Peptides

All peptides were synthesized by automated solid-phase peptide synthesis using the 9-fluorenylmethoxycarbonyl/tert-butyl (Fmoc/tBu) strategy [20] and purified to > 95% homogeneity by preparative HPLC. Analyzation and identification were performed by MALDI-ToF mass spectrometry (Ultraflex III MALDI ToF/ToF, Bruker, Billerica, USA) and reversed-phase HPLC using linear gradients of solvent B (acetonitrile + 0.08% trifluoroacetic acid) in A (H_2_O + 0.01% trifluoroacetic acid).

### Plasmids

The Y_2_R within the pEYFP-N1 expression vector (Clontech) was used for fluorescence microscopy, cAMP-assay, Ca^2+^-assay, IP-one assay and binding assays. An internalization deficient Y_2_ receptor mutant (S^374^T^376^T^379^D-Y_2_R) was generated by QuikChange site-directed mutagenesis (Stratagene) using appropriate primer pairs. The Y_2_R-mutant within C-terminally fused pEYFP-N1 was used for fluorescence microscopy, cAMP-assay, Ca^2+^-assay. For co-transfection experiments Y_1_R within the pEYFP-N1 and MCR1 within the pEYFP-pV2 expression vector were used for cAMP-assay and Ca^2+^-assay. For cAMP readout, pGL4.29[luc2P/CRE/Hygro] encoding for a luciferase reporter gene (luc2P) under the control of the cAMP response element (CRE) was used (Promega). Overexpression of Gα_i2_ was performed by using Gα_i2_-Venus-pcDNA3 vector. Additionally, for measuring receptor activation using the Gα_q_ pathway, the chimeric Gα_Δ6qi4myr_ protein was used (kindly provided by E. Kostenis, Rheinische Friedrich-Wilhelms-Universität, Bonn, Germany) [21]. The identity of all plasmid constructs was verified by Sanger dideoxy sequencing.

### Cell culture

All cell lines were cultured in flasks to confluence prior to use in a humidified atmosphere at 37 °C and 5% CO_2_. HEK293 cells (human embryo kidney) were maintained in Dulbecco’s modified Eagle’s medium (DMEM) with 4.5 g/l glucose and L-glutamine and Ham’s F12 (1:1, Lonza) supplemented with 15% (v/v) heat-inactivated fetal bovine serum (FBS). Stably transfected HEK293-HA-Y_2_R-eYFP were generated by transfecting 13 μg of linearized HA-hY_2_R-eYFP-pVitro plasmid with 20 μl Lipofectamin® 2000 (Invitrogen™) according to the manufacturer’s protocol. For culturing the same conditions as previously described were used and 100 μg/ml hygromycin B gold (Invivogen) was supplied to the medium to ensure stable transfection. SHSY5Y, human bone marrow neuroblastoma cells, derived from a parental SK-N-SH human neuroblastoma cell line [22] were maintained in DMEM with 4.5 g/l glucose and L-glutamine and Ham’s F12 (1:1, Lonza) supplemented with 15% (v/v) heat-inactivated fetal bovine serum (FBS) and 0.1 mM NEAA (Lonza). SMS-KAN cells were cultivated in Eagle’s Minimal Medium (EMEM; Lonza) supplemented with 15% FCS, 4 mM L-glutamine (Lonza) and 0.02 nM nonessential amino acids (NEAA) (Lonza). All cells were kept in a humidified atmosphere at 37 °C and 5% CO_2_.

Short tandem repeat analyses were performed to verify cell line identity, and all cell lines were tested negative for mycoplasma contamination.

### Live cell microscopy

HEK293 or HEK293-HA-Y_2_R-eYFP were re-seeded (150.00/well) into sterile poly-D-lysine covered μ-slide 8 wells (Ibidi) and grown in a humidified atmosphere at 37 °C and 5% CO_2_. For transfection, cells were cultured up to 70–80% confluency and subsequently transfected with 1.0 μg total DNA using Lipofectamine® 2000 transfection reagent (Invitrogen) according to the manufacturer’s protocol. For single transfection in empty HEK293 cells 1.0 μg of HA-Y_2_R-eYFP-N1 plasmid DNA was used. At the experimental day cells were starved in Opti-MEM® reduced serum medium (Gibco®) containing Hoechst33342 (Sigma) for 30 min at 37 °C. LysoTracker®Blue with a final concentration of 1 μM was used for visualization of the lysosomes. Internalization studies were performed by stimulating cells with 1 μM NPY or 100 nM fluorescent NPY derivatives in Opti-MEM® reduced serum medium for 60 min at 37 °C. For recycling studies, cells were washed twice with acidic wash buffer (50 mM glycine, 100 mM NaCl, pH 3.0) and neutralized once with Hank’s balanced salt solution (HBSS; PAA), followed by a recovery period for 60 min in ligand-free media supplemented with 100 μg/ml cycloheximide (CHX; Merck/Calbiochem®) and with or without 20 mM NH_4_Cl as an expression inhibitor and recycling inhibitor respectively. Second stimulation experiments using 5-carboxytetramethylrhodamine (TAMRA)-NPY were performed by stimulating cells in a first round with 1 μM NPY, subsequent acidic wash and either direct stimulation in a 2nd round with 100 nM TAMRA-NPY or incubation in ligand-free media supplied with CHX and with or without NH_4_Cl for 60 min prior to the 2nd round stimulation. All microscopy images were obtained using the AxioObserver.Z1 microscope equipped with an ApoTome imaging system (Zeiss, Jena). Within one experimental setup, all images were taken using a fixed exposure time for the single fluorescence channels.

Microscopy pictures were processed with the standard software Axio vision 4.8 and exported as an 8 Bit TIFF file (Tagged Image File Format). The open access software Image J was applied for the analysis of the pictures. For determination of mean cell surface fluorescence (MCSF) ten nonadjacent cells per image were measured using the segmented line function. Calculation of the relative TAMRA fluorescence, the Raw Intensity Density was measured under distinct conditions. The particular background fluorescence of each image was subtracted after every evaluation. Since this program uses the single gray levels for evaluation, the calculated values were evaluated retroactively as relative fluorescence intensity and analyzed with the statistical program GraphPad Prism.

### Immunostaining

HEK293-HA-hY_2_R-eYFP cells were seeded on poly-D-lysine coated coverslips (500.000 /well). After 24 h incubation under humidified atmosphere at 37 °C and 5% CO_2_, cells were starved in Opti-MEM® reduced serum medium for 30 min, followed by stimulation with 1 μM TAMRA-NPY for 60 min and subsequently washed three times with PBS and incubated in recovery medium for indicated time periods. Gradually preparation of all samples was performed in 10 min intervals of 2% paraformaldehyde (PFA)/PBS on ice, 2% PFA/PBS at RT and finally 4% PFA/PBS at RT. Samples were blocked and permeabilized with 10% BSA/0.1% TritonX-100/PBS for 1 h at RT. Incubation with anti-EEA1 (Santa Cruz, sc-33,585) (1:100) in 1% BSA/0.01% TritonX-100/PBS occurred overnight at 4 °C. The next day, samples were treated with anti-rabbit-AF350 (InvitrogenTM) (1:400) in 1% BSA/0.01% TritonX- 100/PBS for 2 h at RT and mounted with Fluoromount-G®.

### IP-one assay

HEK293 cells were grown in 25 cm^2^ culture flask and co-transfected with 800 ng plasmid encoding the Gα_Δ6qi4myr_ protein and 3200 ng plasmid encoding the Y_2_R fused C-terminally to eYFP applying Metafectene® Pro (Biontex Laboratories GmbH) according to the manufacturer’s protocol. One-day post transfection, cells were seeded (75,000 cells/well) into white poly-D-lysine covert 96-well plates (Greiner Bio-one) and incubated overnight at 37 °C. Receptor activation studies were performed by using the IP-one Gq assay kit (Cis-Bio). Prior to detection of IP- species; cells were either stimulated with buffer, 100 nM or 1 μM NPY for 60 min and washed with acidic wash buffer and HBSS. For recycling, cells were incubated for 60 min with assay buffer, supplemented with 100 μg/ml CHX (Merck/Calbiochem®). After recovery, the recycling medium was removed and 30 μl stimulation solution containing NPY in the concentration range of 10^− 13^ M to 10^− 7^ M and LiCl (inhibition of IP-species degradation) was added and cells were incubated for 1.5 h at 37 °C. Stimulation was stopped by adding lysis buffer supplied with antibody 1 and antibody 2 according to the manufacturer’s protocol. After 60 min of incubation at room temperature, the emission at 620 nm and 665 nm was measured and the ratio (acceptor 665 nm/donor 620 nm) was calculated.

### Ca^2+^-assay

HEK293 cells were grown in 25 cm^2^ culture flask up to 70–80% confluence in a humidified atmosphere at 37 °C and 5% CO_2_. Co-transfection of 800 ng plasmid encoding the Gα_Δ6qi4myr_ protein and 3200 ng plasmid encoding for the Y_2_R fused C-terminally to eYFP was performed by using Metafectene® Pro (Biontex Laboratories GmbH) according to manufacturer’s protocol. One-day post transfection, cells were re-seeded (100.000 cells/well) into a black poly-D-lysine covert 96-well plates (Greiner Bio-one) and incubated overnight at 37 °C. Two-days post transfection Ca^2+^ experiments were performed with the help of the FLIPR® Calcium Assay Kit (Molecular Devices). Cells were stimulated in a first period either with buffer, 10 nM, 100 nM or 1 μM NPY for 60 min, followed by the removal of the peptide solution and subsequent acidic wash two times with acidic wash buffer (50 mM glycine, 100 mM NaCl, pH 3.0) and one neutralization step with Hank’s balanced salt solution (HBSS; PAA). For recycling, cells were incubated for 60 min with assay buffer (20 mM HEPES, 2.5 mM Probenecid in HBSS, pH 7.5) supplemented with 100 μg/ml CHX (Merck/Calbiochem®) and 1 μM Fluor-2-AM staining dye. For measurement, a subset of respective stimulated cells was treated with NPY in a concentration range 10^− 11^ M to 10^− 6^ M and the Ca^2+^-response was detected using the FlexStation (Molecular Devices). The excitation occurred at a wavelength of 485 nm and the emission was measured at 525 nm. The maximum Ca^2+^-response of each concentration was displayed as x-fold over basal in a concentration-response. For the Gα_Δ6qi4myr_ overexpression experiments, the transfection ratio between receptor DNA and G protein DNA was varied from the initial concentration 4:1, using a total DNA amount of 4000 ng. By keeping the G protein amount equal to 800 ng, the resulting discrepancy was filled with pcDNA3.

### cAMP-assay

HEK293 cells were grown in 25 cm^2^ culture flask up to 70–80% confluence in a humidified atmosphere at 37 °C and 5% CO_2_. Co-transfection of 3000 ng pGL4.29[luc2P/CRE/Hygro] (Promega) plasmid and 3000 ng plasmid encoding for the respective receptor was performed using Metafectene® Pro (Biontex Laboratories GmbH) according to the manufacturer’s protocol. One-day post-transfection cells were seeded into white poly-D-lysine covert 96-well plates (Greiner Bio-one) and incubated overnight at 37 °C. For pertussis toxin experiments, medium was supplied with certain concentration (100 ng/ml, 250 ng/ml, 500 ng/ml) of pertussis toxin (Sigma). Two-day post-transfection cAMP experiments were performed with the help of ONE-Glo substrate (Promega), allowing luminescence measurement with the Tecan Infinite M200. Cells were stimulated in a first period either with buffer, 10 nM, 100 nM or 1 μM NPY for 60 min, followed by the removal of the peptide solution and subsequent wash two times with acidic wash buffer (50 mM glycine, 100 mM NaCl, pH 3.0) and one neutralization step with Hank’s balanced salt solution (HBSS; PAA). For recycling, cells were incubated for 60 min with DMEM supplemented with 100 μg/ml CHX (Merck/Calbiochem®). For cAMP measurement, a subset of respectively stimulated cells was treated with a mixture of NPY in a concentration range 10^− 12^ M to 10^− 6^ M and 5 μM forskolin and incubated for 4 h at 37 °C. As a positive control only 5 μM forskolin were added to the wells. After incubation, medium was exchanged by 30 μl fresh DMEM (RT). 30 μl ONE-Glo substrate was added and after 5 min of incubation the cAMP level was measured by detecting the luminescence. For co-transfection experiments the transfection of two receptors simultaneously and pGL4.29[luc2P/CRE/Hygro] plasmid were performed in a ratio 1:1:1, keeping the total amount of 6000 ng. For Y_1_/Y_2_ co-transfection, cells were treated with either 1 μM F^7^P^34^-NPY or Ahx^5–24^NPY first, followed by the washing step and the recovery period. The second stimulation was performed in a peptide concentration range, addressing the respective receptor that was missed in the first round. Procedure was analogous concerning co-transfection with melanocortin 1 receptor, first stimulation was performed either with 1 μM NPY or 1 μM NAPamide.

Endogenous Y_2_ receptor activation was performed in SMS-KAN cells by using the Gs dynamic assay kit (Cis-Bio). Prior to detection of cAMP concentration, SMS-KAN cells were grown in 12 well plates in a humidified atmosphere at 37 °C and 5% CO_2_. At the experimental day, cells were stimulated first either with buffer (control cells) or 1 μM NPY for 60 min, followed by the removal of the peptide solution and subsequent washing steps with acidic wash buffer twice (50 mM glycine, 100 mM NaCl, pH 3.0) and one neutralization step with HBSS (PAA). For recycling, cells were incubated for 60 min with DMEM supplemented with 100 μg/ml CHX. For cAMP measurement, the cells were detached from the 12-well plate by adding 150 μl stimulation buffer and gently using a cell scraper. Finally, a subset of 50.000 cells in an end volume of 5 μl was reseeded in a 384 well plate. Cells were treated with a mixture of NPY in a concentration range of 2 × 10^− 12^ M to 2 × 10^− 5^ M (final concentration 10^− 12^ M to 10^− 5^ M) and 10 μM forskolin (final concentration 5 μM) simultaneously and incubated for 1 h at 37 °C. As a positive control only 5 μM forskolin (final concentration) was added to the wells. Stimulation was stopped by adding 5 μl of cAMP-d2 and cAMP-cryptate working solution diluted in lysis- and detection buffer to each well and incubated at room temperature for another 60 min according to manufacturer’s protocol. After 60 min of incubation at room temperature, the emission at 620 nm and 665 nm was measured and the ratio (acceptor 665 nm/donor 620 nm) was calculated.

### Specific radioligand binding assay

HEK293 cells were maintained in 25 cm^2^ culture flask up to 70–80% confluency in a humidified atmosphere at 37 °C and 5% CO_2_. Cells were transfected with 4000 ng plasmid encoding for the Y_2_R using Metafectene® Pro (Biontex Laboratories GmbH) according to the manufacturer’s protocol. For Gα_i_-overexpression experiments cells were transfected with 3000 ng plasmid encoding for the Y_2_R and 1000 ng plasmid encoding for the Gα_i2_-subunit. One-day post-transfection cells were seeded into poly-D-lysine covert 48-well plates (Greiner Bio-one) and incubated overnight at 37 °C. Two-day post-transfection cells were treated either with buffer or 1 μM NPY for 60 min subsequently washed twice with acidic wash buffer and once with HBSS, followed by a recovery period in ligand free medium containing 100 μg/ml CHX (Merck/Calbiochem®). After treatment, cells were immediately cooled down on ice, washed once with PBS and incubated with 6 × 10^− 11^ M human [^125^I]-PYY in binding buffer for 4 h. Binding buffer consisted of OptiMEM, 50 mM Pefabloc® SC, 1% BSA and for replacement NPY in a concentration range 10^− 11^ M to 10^− 6^ M. After incubation, cells were washed twice with ice-cold PBS and lysed with 0.2 M NaOH. Lysates were transferred into scintillation cocktail and radioactivity was detected with the help of Microbeta2™ counter.

### Statistical analysis

Calculations of means, S.E.M. and statistical analysis were performed using PRISM 5.0 program (GraphPad Software, San Diego, USA). Significances were calculated according to one-way ANOVA and Tukey’s or paired, two-tailed t-test.

## Results

### Y_2_R and NPY co-internalize but underlie different trafficking routes

**Fig. 1:**
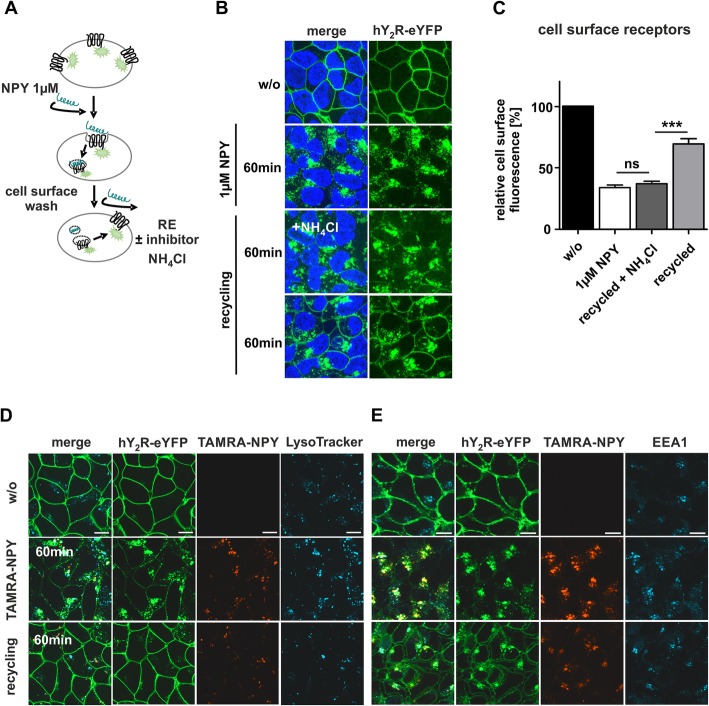
hY_2_R recycles back to the membrane, while the peptide is degraded. **a** Scheme of hY_2_R internalization and recycling experiment quantifying cell surface receptors. **b** Live cell images of HEK293 cells transiently transfected with HA-Y_2_R-eYFP. Receptor (green) localization was determined by fluorescence microscopy prior (w/o) and after stimulation with 1 μM of the endogenous ligand NPY at 37 °C, subsequent washing and incubation in ligand free medium supplemented with 100 μg/ml CHX in a 60 min recycling period. **c** Quantification of cell surface fluorescence intensity using Image J. Cell surface receptors before stimulation is set to 100% (w/o, black bar). Stimulation with 1 μM NPY reduced the amount of membrane receptors (white bar), which increased again after the recycling period (light grey). However, NH_4_Cl- treated cells displayed no reappearance of hY_2_R back to the membrane (dark grey). **d** Live cell images of HEK293-HA-hY_2_R-eYFP cells stained with 1 μM LysoTracker®Blue (blue). Subsequent incubation with 100 nM TAMRA-NPY (red) for 60 min at 37 °C leads to a rapid co-localization of hY_2_R and peptide (yellow) in early endosomes (EE1A, blue) after immunostaining (**e**). The receptor was separated during a 60 min recycling period and transported back to the membrane, whereas TAMRA-NPY was co-localized with the lysosomal marker (light blue). Scale bar: 10 μm, experiments represent data *n* ≥ 3; significance was determined by one-way ANOVA, Tukey post test, ns: not significant, ***: *P* < 0.0001

Understanding regulatory mechanisms such as desensitization is fundamental to understand the endocytotic and post-endocytotic fate of a receptor. Previous investigation clearly demonstrated that human Y_2_R internalizes by arrestin-dependent and independent mechanisms following high agonist concentration. Arrestin 3 is fully engaged by the C-terminus of the receptor and the transmembrane core, thus physically blocking G protein interactions and terminating signaling. Once internalized, the receptor re-appears at the cell membrane after agonist washout. To get more detailed information about the cellular transport mechanism, we first examined the post-endocytotic sorting of the receptor and its agonist NPY. We used a stably transfected HEK293 cell line expressing the Y_2_R N-terminally fused to hemagglutinin (HA), and carrying enhanced yellow fluorescent protein (eYFP) at its C-terminus. Enabled by the YFP-labeling of the receptor and an additional TAMRA-NPY variant, live cell fluorescence microscopy was applied to study the intracellular fate of the receptor after agonist stimulation as graphically illustrated in Fig. 1a. As shown in Fig. 1b, the receptors are expressed predominantly in the plasma membrane prior to agonist stimulation. For quantification, the mean cell surface fluorescence (MCSF) was determined and set to 100% (Fig. 1c, w/o). Stimulation of HEK293-HA-Y_2_R-eYFP with 1 μM NPY led to strong internalization and accumulation into intracellular compartments (33 ± 2%, MCSF, white bar). Removing the stimulation solution and incubation in ligand free medium for 60 min resulted in a reappearance of receptor at the plasma membrane up to 69% (SEM ± 4%, light grey bar), which was inhibited by adding 20 mM NH_4_Cl as recycling inhibitor. Co-internalization of the ligand-receptor-complex was observed by incubation with TAMRA-NPY (Fig. 1d/e). Immunostaining of the early endosome antigen EE1A displayed an intracellular accumulation of TAMRA-NPY in early and sorting endosomes, prior to further distribution (Fig. 1e). After ligand removal and during the recycling period, the amount of co-localized NPY-receptor-complexes decreased in early endosomes. While the receptor fluorescence at the cell membrane increased, the total amount of red peptide fluorescence decreased. Using a lysosomal stain, the reduction of peptide fluorescence was identified as a result of degradation in lysosomes (Fig. 1d). Accordingly, the Y_2_R and its ligand pass through different intracellular trafficking routes. While the receptor recycles back to the plasma membrane, and should be available for a further activation, the ligand is degraded in lysosomes.

### Reduced Y_2_R internalization in second stimulation experiments despite recycling

**Fig. 2:**
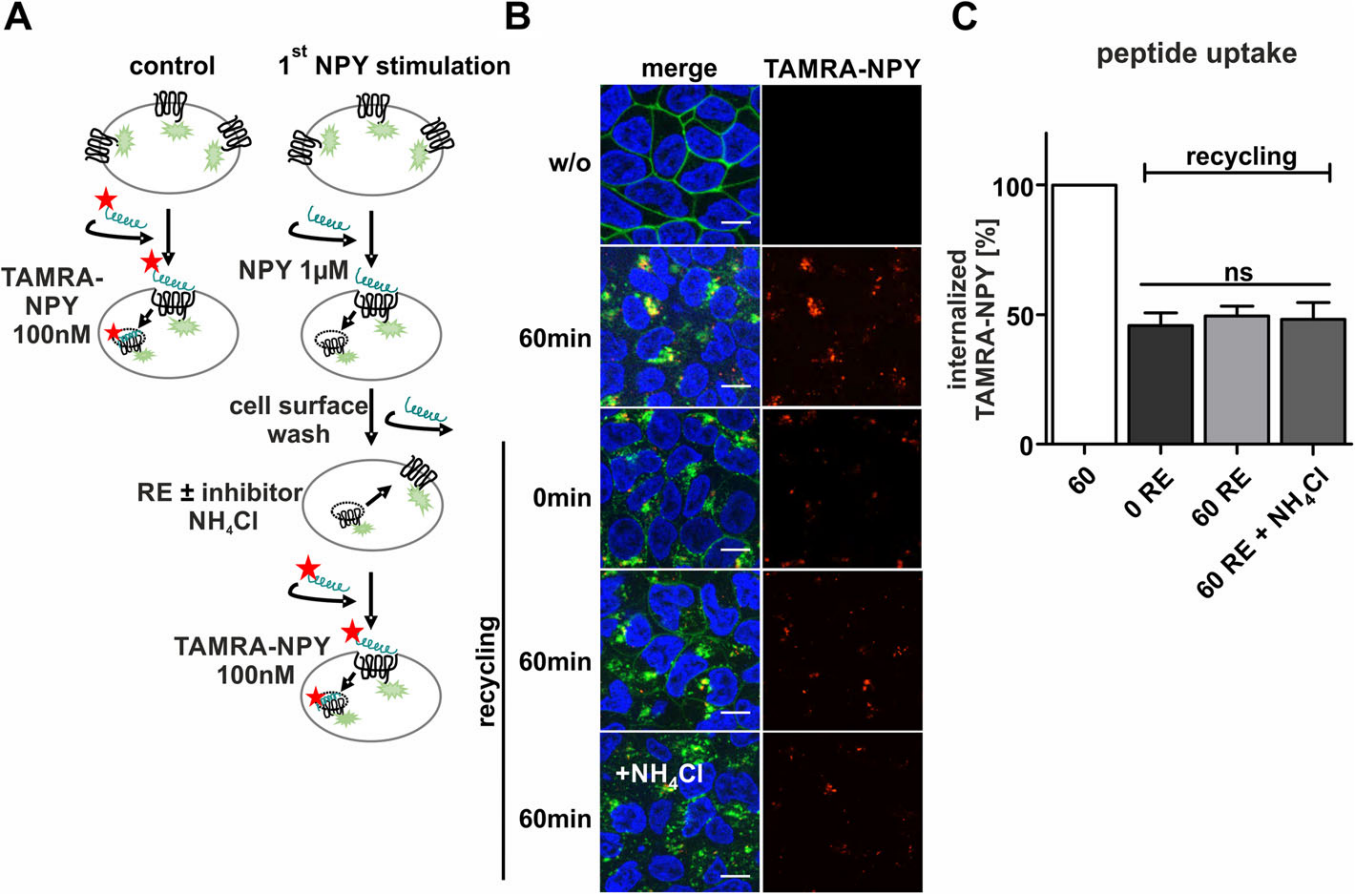
hY_2_R exhibit impaired internalization properties after recycling. **a** Scheme of hY_2_R internalization- and recycling experiment quantifying internalized peptide. **b** Live cell images of HEK293-HA-hY_2_R-eYFP and TAMRA-NPY uptake at distinct conditions. Control cells (60 min) were treated with 100 nM peptide for 60 min at 37 °C. Stimulation with 1 μM NPY, washing and direct second stimulation (0 min RE) with 100 nM TAMRA-NPY for 60 min, second stimulation after a 60 min recycling period in the presence or absence of the recycling inhibitor NH_4_Cl (60 min RE ± NH_4_Cl). Ribosomal protein synthesis was inhibited by cycloheximide [CHX]. The nuclei were stained with Hoechst33342 (blue). **c** Quantification of TAMRA-NPY uptake. Uptake of HEK293-HA-hY_2_R-eYFP cells after stimulation with 100 nM peptide for 60 min at 37 °C was set to 100% (w/o, white bar). Stimulation with 1 μM NPY, washing and direct second stimulation (0 RE, dark grey bar) or after a recycling period ± NH_4_Cl (60 RE ± NH_4_Cl) with 100 nM TAMRA-NPY for 60 min revealed no difference in TAMRA internalization. Scale bar: 10 μm, experiments represent data *n* ≥ 3; significance was determined by one-way ANOVA, Tukey post test, ns: not significant, ***: *P* < 0.0001

Next, we aimed to confirm the functionality of the recycled receptors by testing whether recycling represents a quick resensitization and qualifies the system as a suitable drug-shuttle. Thus, we examined the internalization properties in a second period of stimulation, which is summarized in a graphical scheme in Fig. 2a. Ligand uptake of HEK293-HA-Y_2_R-eYFP was measured by Raw Intensity Density using Image J. First stimulation of cells with 100 nM TAMRA-NPY resulted in high yields of internalized ligand after 60 min (Fig. 2b), which was set to 100% as control (Fig. 2c). Stimulation of the cells with 1 μM non-fluorescent NPY, intensive acidic wash and subsequent stimulation with 100 nM TAMRA-NPY led to an expected significantly reduced TAMRA-NPY uptake (45 ± 5%, 0 RE), due to receptor internalization and reduced cell surface receptor amounts. However, the peptide uptake was similar and not increased when the receptor was allowed to recycle after extending the recovery period up to 60 min (49 ± 4%, 60 RE). Next, the uptake of TAMRA-NPY was measured in presence of the recycling inhibitor NH_4_Cl during recovery period. No further reduction of internalized peptide was detectable (48 ± 46%, 60 RE + NH_4_Cl), confirming that the recycled receptors do not contribute to peptide uptake into the cell. This indicates a significantly reduced functionality of the recycled receptors with respect to internalization.

### Y_2_R stimulation but not internalization leads to sustained reduction of cAMP response

**Fig. 3:**
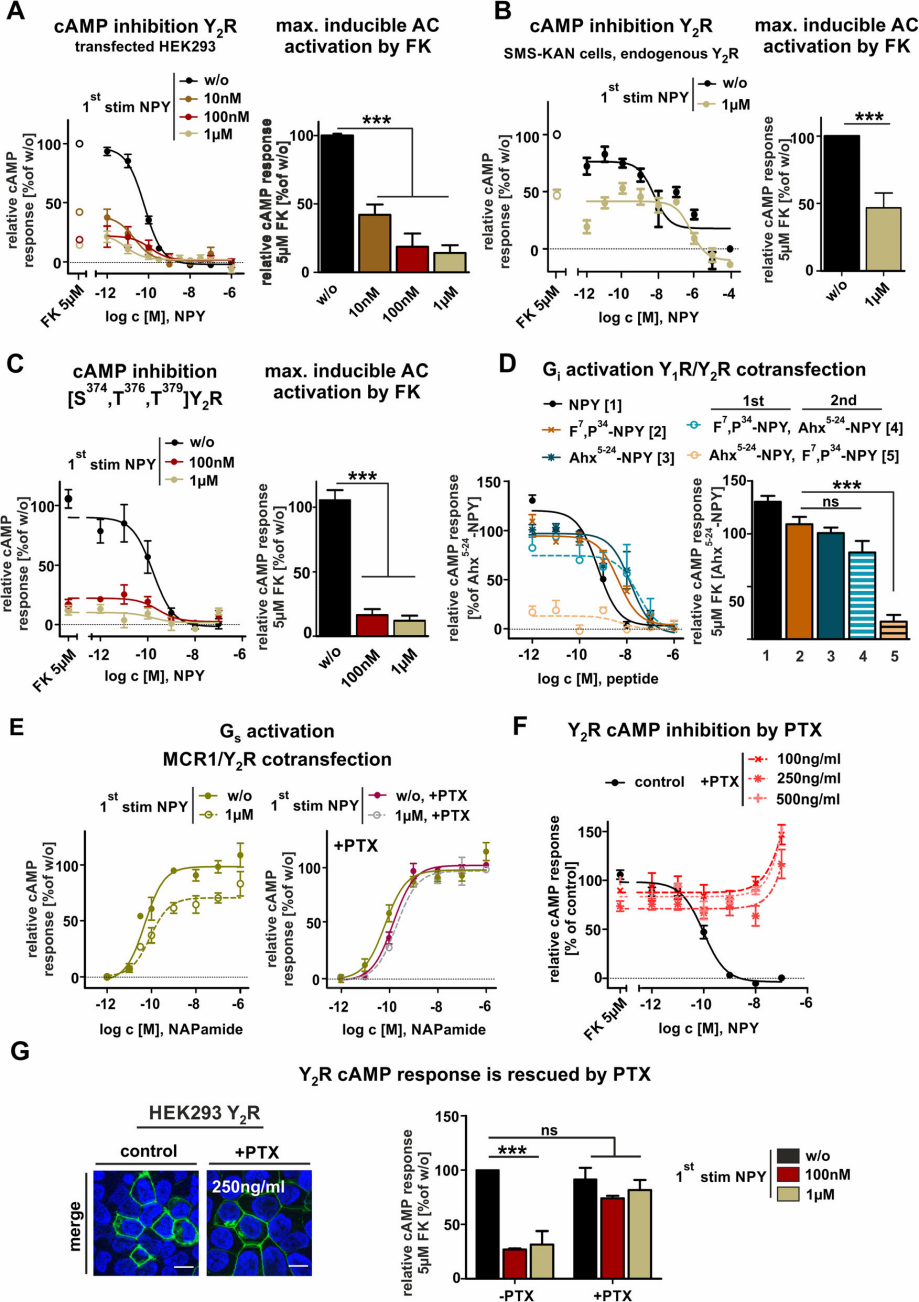
Sustained inhibition of the adenylyl cylase (AC) by Y_2_R stimulation can be abolished by PTX-treatment. **a** Concentration-response curves of transiently transfected HEK293-hY_2_R were measured by cAMP–accumulation assay without first stimulation (w/o; black line) and after stimulation with 10 nM, 100 nM and 1 μM NPY, respectively (brown, red, olive-green). AC inhibition caused by Gα_i_ activity was measured in the presence of 5 μM forskolin. The maximum cAMP response is reduced in stimulated cells. **b** Data were verified by testing native Gα_i_-signaling with SMS-KAN cells that endogenously express Y_2_R. We observed the same reduction of the maximal inducible adenylyl cyclase response induced by forskolin in stimulated cells (olive-green). Data were validated with **c**) an internalization deficient mutant S^374^T^376^T^379^D-Y_2_R. **d** Concentration-response curves of transiently co-transfected HEK293-hY_2_R/hY_1_R were measured by cAMP–accumulation assay. Cells without first stimulation (NPY, black line; F^7^, P^34^-NPY, orange line; Ahx^5–24^-NPY, blue line) or with first stimulation with 1 μM F^7^, P^34^-NPY, a selective Y_1_R peptide (FP), followed by stimulation with Ahx^5–24^-NPY, a selective Y_2_R agonist (dashed blue line) exhibited no significant loss in AC-activity. First stimulation with 1 μM Ahx^5–24^-NPY, followed by stimulation with F^7^, P^34^-NPY (FP, dashed orange line) revealed an obvious inhibition of AC-activity. Data were normalized to Ahx^5–24^-NPY as control curve (100%, blue line). **e** Concentration-response curves of transiently co-transfected HEK293-hY_2_R with melanocortin 1 receptor were measured by cAMP–accumulation assay and had only slight impact on Gα_s_-signaling, as the inhibitory effect of Y_2_R is still present after stimulation with 1 μM NPY (left panel) but is completely abolished in presence of PTX and thus preventing Gα_i_- signaling (right panel). **f** cAMP-accumulation assay of transiently transfected HEK293-hY_2_R treated with varying concentrations of pertussis toxin (PTX) caused inhibition of AC-activity compared to control curve (black line). **g** AC-activity is rescued comparing ± PTX- treated cells after first stimulation with different NPY concentration and reached again the cAMP level of control (w/o). Experiments represent data *n* ≥ 3; significance was determined by one-way ANOVA, Tukey post test, ns: not significant, ***: *P* < 0.0001, **: *P* < 0.001

To clarify whether the diminished endocytosis of recycled receptors is due to impaired arrestin recruitment and thus specific to endocytosis or rather a consequence of reduced receptor functionality in general, we investigated receptor activation in a next step by analyzing cellular cAMP levels (Fig. 3). Without NPY-stimulation, 5 μM of the adenylyl cyclase activator forskolin typically increases the baseline of intracellular cAMP concentration in Y_2_R-expressing HEK293 cells by ~ 10 fold (set to 100%), which is completely reversed by stimulation with NPY with an EC_50_ of 0.04 nM. Surprisingly, the maximal inducible cAMP response, induced by forskolin was dramatically reduced in cells with recycled Y_2_R (Fig. 3a). Stimulation with 100 nM or 1 μM NPY followed by agonist washout and 60 min recycling period (same procedure as used in microscopy experiments) led to a drop of cellular cAMP in response to 5 μM forskolin (18 ± 5%, 14 ± 9% respectively, Fig. 3a). Even stimulation with 10 nM NPY already resulted in ~ 60% reduction of the susceptibility of AC to Forskolin (41 ± 7%, Fig. 3a, brown curve). Moreover, we have confirmed our findings in a biologically relevant system by investigating the cellular cAMP concentration in SMS-KAN cells that have been isolated from a primary human brain tumor and endogenously express the Y_2_ receptor [23]. Interestingly, we observed the same pattern of sustained Gα_i_-signaling and diminished cellular activatability after stimulation with 1 μM NPY in this transfection-free cellular model (Fig. 3b). The maximally inducible cAMP response with 5 μM forskolin was also significantly reduced in cells with recycled Y_2_R and did not reach the bottom-line of control cells.

Next, we were interested whether this behavior is internalization dependent. Fluorescence microscopy experiments of Y_2_R cells stimulated with 10 nM NPY displayed no internalization at this concentration (Figure S1), indicating an internalization independent mechanism. To confirm this hypothesis, we generated an internalization deficient mutant, exchanging the important phosphorylation residues [S^374^, T^376^, T^379^] to aspartate within the C-terminus [18]. This Y_2_R variant exhibited the same loss of activity as the wild type (Fig. 3c), excluding cellular processes accompanying receptor endocytosis as a cause of these findings. Next, we co-transfected cells with Y_1_R and Y_2_R simultaneously and compared the effects of receptor stimulation. The use of selective peptide agonists allows the addressing of the distinct receptors. Stimulation with either the receptor selective agonist for Y_1_R F^7^, P^34^-NPY or for Y_2_R Ahx^5–24^-NPY resulted in a robust receptor activity with EC_50_-values in the nanomolar range (F^7^, P^34^-NPY: 4.4 nM, Ahx^5–24^-NPY: 14 nM, orange and dark blue line respectively), demonstrating the functionality of the assay setup. By stimulation with 1 μM F^7^, P^34^-NPY to address Y_1_R, followed by stimulation with the Y_2_R selective Ahx^5–24^-NPY after 60 min recovery period, no significant changes in the forskolin-inducible cAMP signal or receptor potency (Fig. 3d, E_max_ 74%, EC_50_ 28 nM, light blue dashed line) was observed. However, addressing the Y_2_R first by using 1 μM Ahx^5–24^-NPY and second stimulation with the Y_1_R selective agonist resulted in a significant loss of forskolin-inducible cAMP (Fig. 3d, E_max_ 13% orange dashed line). As an additional control, we used melanocortin 1 receptors (MCR1), which endogenously couple to Gα_s_-protein, to test the influences on other downstream signaling pathways. Melanocortin receptors are robustly activated by NAPamide, a modified MCR1 agonist, also after stimulation of co-transfected Y_2_R with 1 μM NPY. The maximal cellular cAMP levels however, were slightly reduced by ~ 17% (Emax 83 ± 10%, green dashed line) compared to the control without Y_2_R stimulation, indicating that the inhibitory effects of Y_2_R on cellular cAMP are still present (Fig. 3e). These data suggest a Y_2_R-specific Gα_i_-mediated mechanism, independent of other Gα-signaling pathways. To confirm this, pertussis toxin (PTX) was used, a Gα_i_ sensitive exotoxin that prevents interaction with the G protein and its receptor [24, 25]. Different concentrations were tested first probing the optimal in vitro condition (Fig. 3f). All further experiments were performed using 250 ng/ml PTX. Cell toxicity was examined by microscopy studies and excluded, since no differences in cell morphology were observed (Fig. 3g, images). The presence of PTX clearly rescued the maximal cAMP response induced by forskolin after treatment of cells with NPY (Fig. 3g). Furthermore, applying PTX also on MCR1/Y_2_R co-transfected cells supported Gα_i_-specific effects by abolishing the slight inhibitory effect, seen in the experimental setup, when Y_2_R was stimulated with NPY first (Fig. 3e, right panel). These data demonstrate that Y_2_R stimulation and activation leads to a long-lasting inhibition of Gα_i_-activated effector proteins and consequently downstream in the Gα_i_-pathway.

### High G protein turnover of Y_2_R is terminated by depleted intracellular G protein pool

**Fig. 4:**
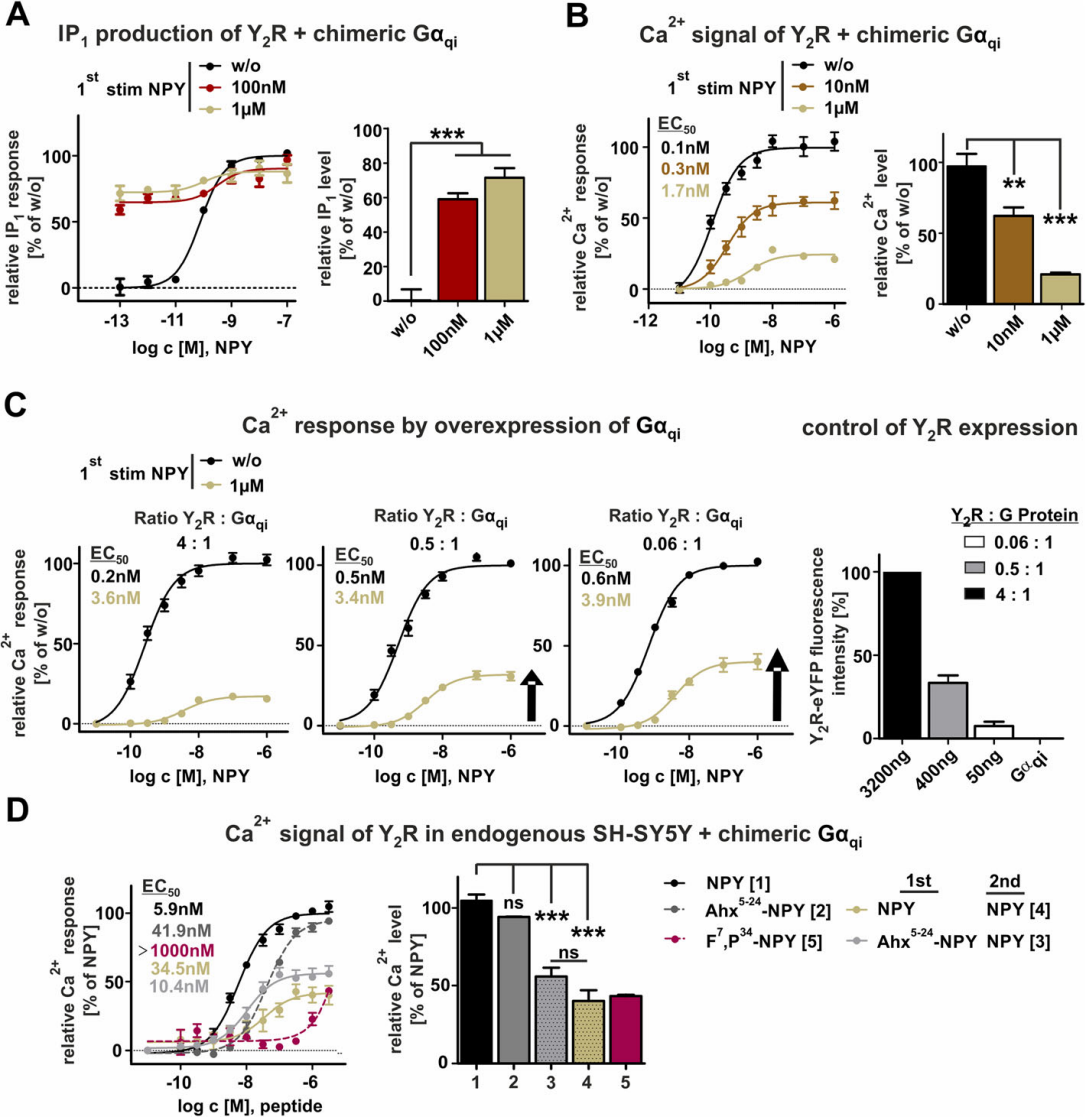
Gα_q_ signal transduction is reduced but G protein overexpression counteracts the effect in HEK293 cells. **a** Concentration-response curves of transiently co-transfected HEK293-hY_2_R + Gα_qi_ were measured by IP–accumulation assay, using the chimeric G protein as a tool. Prolonged IP signal was observed after first stimulation with 100 nM (red) or 1 μM (olive-green), subsequent washing and 60 min recover of receptor in ligand free media compared to control curve without stimulation (w/o, black). **b** Decreased Ca^2+^ response from transiently transfected HEK293-hY_2_R-eYFP cells was obtained after stimulation with either 10 nM (brown) or 1 μM (olive-green) for 60 min at 37 °C followed by subsequent washing and 60 min recycling. **c** Overexpression of G protein and modifying the ratio between receptor and G protein led to a partial gain of Ca^2+^ signal after stimulation with 1 μM NPY, followed by washing step and 60 min recovery. Fluorescence intensity was investigated to quantify the receptor-eYFP fusion protein. **d** Data were confirmed by testing Gα_qi_-signaling with SH-SY5Y cells that endogenously expressing Y_2_R, and were transiently transfected with the chimeric G_qi_ protein. We observed an attenuated Ca^2+^-response for receptors stimulated either with 1 μM NPY (olive-green) or 1 μM Ahx^5–24^-NPY (light grey), a selective Y_2_R agonist, and stimulated with NPY in a second experiment. Treatment with F^7^, P^34^-NPY, a selective Y_1_R agonist (berry) proved Y_2_R specificity. Experiments represent data *n* ≥ 3; significance was determined by one-way ANOVA, Tukey post test, ns: not significant, ***: *P* < 0.0001

These data raise the question, whether this sustained inhibition of Gα_i_-pathway is due to a specific and exceptionally tight interaction between Y_2_R-activated Gα_i_-protein and the adenylyl cyclase (AC) or whether a very high G protein turnover and thus high number of active Gα_i_-GTP is responsible for the phenomenon. We took advantage of the chimeric Gα_Δ6qi4myr_ protein (Gα_iq_) as a tool system. This chimeric Gα-protein couples to G_i_-preferring receptors, but addresses cellular effectors of the Gα_q_-pathway and activates phospholipase C, which is directly measurable by the increase of cellular inositol phosphate (IP) and Ca^2+^influx [21]. Moreover, the chimeric G protein is co-transfected with the receptor, whereby modulation of both receptor and G protein expression levels and ratio by transfection provides an excellent assay setup. First, we tested receptor activity using both IP accumulation assay and Ca^2+^-assay, as a live imaging assay, since the Gα_qi_ re-routes the native Gα_i_-pathway to the phospholipase pathway. Both assay setups were suitable for measuring receptor activity and showed similar EC_50_ –values compared to the endogenous signaling pathway detected with cAMP accumulation assay. In accordance with our previous results, the measured activity of stimulated and recycled receptors displayed an impaired functionality comparable to cAMP accumulation assay (Fig. 4a/b).

For the IP accumulation assay, we first stimulated the Y_2_R with either 100 nM or 1 μM NPY, followed by an extensive washing step and recovery period in ligand free medium. All pre-treatment and recovery steps were conducted in the absence of lithium chloride to ensure normal cellular IP turnover. Nonetheless, we found a sustained IP-response, since the initial IP-level for NPY stimulation is still at 60% and does not reach the basal IP-level after recovery period again (Fig. 4a, red curve/bar). This effect is even higher for stimulation with 1 μM NPY (Fig. 4a, olive curve/bar). These results support our findings of a sustained Gα-GTP activity once activated by the Y_2_R. Furthermore, it suggests this effect to be caused by high G protein turnover of Gα_i_ and Gα_qi_ and not to be limited to Gα_i_ and AC interaction.

In the next step, we performed Ca^2+^-measurements downstream of the chimeric Gα_qi_-protein. Beside the fast rise of intracellular Ca^2+^-levels, an advantage of measuring Ca^2+^-influx after receptor stimulation is the rapid decrease and resetting to the basal Ca^2+^-levels. Thus, the increased cellular Ca^2+^ levels after the first stimulation remain “invisible” and the measurements give access to the actual amount of G protein activation after the receptor recovery period. The baselines of control and after the first round of stimulation start at a comparable intensity contrary to IP-one accumulation assay and thereby the assay window is magnified.

Stimulating cells in a concentration-response range resulted in a robust receptor activity response curve with EC_50_-values in the nanomolar range (Fig. 4b). Interestingly, also in this transient measurement, we found a loss of activity after the first stimulation. Stimulation first with 10 nM NPY resulted in 40% reduced Ca^2+^-influx (Fig. 4b, 62% ± 6), which is even more reduced after stimulation with 1 μM (Fig. 4b, 21% ± 1%). Taken together our findings suggest that a very high G protein turnover of the NPY-activated Y_2_R depletes the functional cellular G protein pools, limiting signaling responses even after agonist washout and a recovery period of 1 h. To clarify whether a longer recovery period might improve Y_2_ receptor functionality after the first stimulation we also modified and prolonged the recycling period up to 6 h. However, even under these conditions the signal did not exceed 50% of control cells, which further underlines the strong and persistent downregulation of receptor activity (Figure S2).

To corroborate our hypothesis, we altered the ratio of receptor to G protein in the cell. Relative overexpression of Gα_Δ6qi4myr_ leads to a partial rescue of the receptor activation after receptor recycling (Fig. 4c, olive green lines). Expression levels of the receptors were quantified by fluorescence measurements using the C-terminally fused YFP and are displayed in Fig. 4c (right panel). Only Gα_qi_ transfection served as negative control, while the commonly used condition 4:1 (3200 ng receptor: 800 ng G protein) was set to 100%. Even under conditions with 17-fold excess of G protein at DNA level (0.06:1) and a receptor-eYFP fluorescence minimally over untransfected control, the maximal signal in the second stimulation with NPY (olive green lines) did not exceed 50% of control (black lines). However, an EC_50_-shift was also observable, comparing to control receptors (black line) in different transfection conditions, suggesting a sensitive assay-system with a high number of reserve receptors. By reducing the amounts of the receptors by decreased plasmid transfection the number of possible cell surface signaling receptors decline, which is reflected in the rightward shift of the concentration-response-curve. Thus, this experiment clearly demonstrates that the availability of the G protein becomes the limiting factor after Y_2_R stimulation in the second receptor activation. We further excluded that cellular Ca^2+^stores are the limiting factor, as i) Y_2_R activation mainly triggers Ca^2+^-influx from extracellular compartments rather than from cellular compartments (Figure S3A) and ii) stimulation in Y_2_/Y_4_ co-expression systems displays no signal reduction of PP (endogenous Y_4_R ligand), but a strong loss for NPY by adressing the Y_2_R. (Figure S3B).

To confirm this mechanism in a more biologically relevant setting, we transferred the Ca^2+^-influx experiments to human SH-SY5Y cells, which are derived from a parental SK-N-SH human neuroblastoma cell line and endogenously express the neuropeptide Y_2_R [22, 26]. In a first step, we aimed to verify selective Y_2_R signaling by treating the cells either with Y_2_R specific agonist Ahx^5–24^-NPY or Y_1_R specific agonist F^7^, P^34^-NPY. Stimulation of the cells with the selective Y_2_R agonist Ahx^5–24^-NPY resulted in a robust receptor activation (Fig. 4d, dark grey curve, EC_50_: 41.9 nM), whereas no Ca^2+^-signal was detectable when the cells were treated with selective Y_1_R agonist F^7^, P^34^-NPY (Fig. 4d, berry curve, EC_50_ > 1000). In addition to Y_1_ and Y_2_ receptors, NPY can also signal through the related Y_4_ and Y_5_ receptors. To confirm exclusive Y_2_R expression in SH-SY5Y cells we used PP as Y_4_ receptor subtype and [Ala^31^, Aib^32^]-NPY as Y_5_ receptor subtype selective ligands, and investigated the activity of these peptides in Ca^2+^-flux assays [27, 28]. In agreement with literature [22, 26], these peptides displayed no activity in the endogenous background of SH-SY5Y cells (Figure S4).

As already observed in HEK293 cells, we detected a similar loss in activity of endogenous Y_2_R after the first stimulation with both 1 μM NPY (olive green line/bar, E_max_: 40 ± 7%, EC_50_: 34 nM,) and 1 μM Ahx^5–24^-NPY (light grey line/bar, E_max_: 56 ± 6%, EC_50_: 10 nM) respectively. Thus, we demonstrate that the Y_2_R specific effects on intracellular signaling after a first stimulation are not due to the overexpression in commonly used cell lines, but similarly can be found in a biological context.

### Different ligand affinity states serve as feedback mechanism and control Y_2_R signaling

**Fig. 5:**
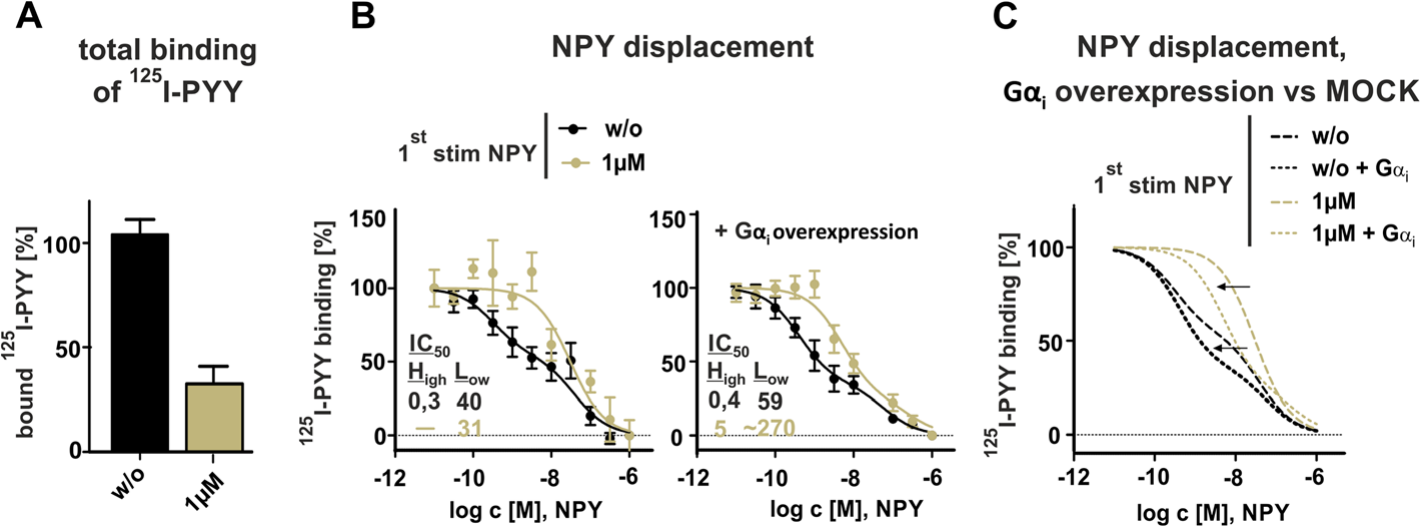
NPY displacement revealed biphasic characteristics and overexpression of Gα_i_ shifted receptors towards the high-affinity state. **a**^125^I-PYY binding studies in transiently transfected Y_2_R HEK293 cells after incubation with 1 μM NPY, acidic wash and receptor recovery revealed diminished binding capacity of recycled receptors (*n* ≥ 3). **b** Specific ^125^I-PYY ligand displacement experiments with transiently transfected Y_2_R HEK293 cells showed a biphasic binding mode with a high affinity state (black line), which is lost after the first stimulation with 1 μM NPY, followed by acidic wash and recovery in ligand free medium for 60 min (olive-green line). For normalizing the data, total binding and unspecific binding of each condition were constrained to 100 and 0% respectively. Overexpression of Gα_i_ shifted the percentage of the receptor towards the active state, which is summarized and represented in **c**). Experiments represent data *n* ≥ 3

Allosteric modulation and regulation is a natural principle to modify the activity of molecules and enzymes in cell signaling. The strength of attraction between a receptor and its ligand – defined as affinity – is crucial for transducing signals. Strong allosteric effects of G protein binding for ligand affinity have been suggested before [29]. Hence, we investigated whether changes in the cellular G protein pool are reflected in the binding properties. To assess ligand-receptor affinity and compare Y_2_R binding prior (w/o, black line) and after stimulation with 1 μM NPY (olive green line), we performed specific [^125^I]-PYY competition radioligand binding assay using transiently transfected Y_2_R in HEK293 cells. The following experiments were performed on ice to prevent receptor internalization and thus allow for equilibrium binding. Specific binding of control receptors that were incubated with buffer represents 100% of possible binding sites (Fig. 5a, black bar). Interestingly, ligand binding of recycled receptors, treated with 1 μM NPY for 60 min, followed by acidic wash and recovery in ligand free medium for 60 min still showed a significant reduction in B_max_ (Fig. 5a, olive green bar, 32 ± 8%), contrary to our observation in microscopy studies that revealed a recycling rate approximately up to 70%. We attribute this to experimental limitations due to the low radioligand concentration compared to the micromolar concentration of peptide used for stimulation. For normalization, total binding and unspecific binding of each condition were constrained to 100 and 0% respectively. NPY displacement experiments show a biphasic binding mode with a high affinity and a low affinity state (Fig. 5b, left panel, w/o black line), which is slightly more pronounced compared to the experiments with isolated membrane preparations [29]. Strikingly, the high affinity state was lost after first stimulation with 1 μM NPY (Fig. 5b, left panel, olive green line). While evaluating the two site fit calculations, control receptors clearly distribute into a high affinity state with a percentage of 45% and an IC_50[high]_ of 0.3 nM and a low affinity state with an IC_50[low]_ of 40 nM. Analyzing recycled receptors, the calculated IC_50[high]_ and IC_50[low]_ is 30 nM and comparable when applying one site fit (IC_50_ 35 nM, data not shown), indicating an impaired ligand affinity due to the loss of a high affinity receptor conformation. To address the question, whether the affinity shift is a result of G protein depletion, we co-transfected Gα_i2_ with the Y_2_R (Fig. 5b, right panel). In consequence of the overexpression, an increase in the proportion of the high affinity state already for the control receptors were observable (Fig. 5b, right plot, w/o, black line, fraction _[high]_: 65 ± 7%, IC_50[high]_: 0.4 nM, IC_50[low]_: 59 nM). Moreover, the binding properties of recycled receptors were altered positively (Fig. 5b, olive green line). Applying the two site fit model under Gα_i_ overexpressed condition subdivided also recycled receptors in a high-affinity state [IC_50[high]_: 5 nM] with a considerable share of 75 ± 19% (fraction_[high]_) and only a minor fraction of receptors in a low affinity state with a calculated IC_50[low]_ of 270 nM. Taken together, overexpression of Gα_i_ shifts the percentage of the receptor towards the active state as shown in Fig. 5c. Thus, the binding experiments support our finding that the G protein pool is depleted after Y_2_R stimulation, which limits further receptor-ligand binding and downstream cellular signaling.

## Discussion

Understanding the regulatory mechanisms that control the interplay of receptor activation, receptor trafficking and signal transduction are key processes for the development of new therapeutic approaches. Interestingly, the underlying regulatory processes reveal no consistent scheme and can affect receptor sensitivity in a variety of ways [6, 30, 31]. The classical ligand-induced activation of GPCR is divided into three major steps and can shortly be summarized as i) ligand binding and stabilization of active receptor conformations, ii) binding and activation of the G protein and iii) phosphorylation of the activated receptor targeting for desensitization [32]. The latter protects the cells against chronic and acute overstimulation by fine tuning receptor sensitivity upon external stimuli [33, 34]. Since the neuropeptide Y_2_ receptor is involved in multiple physiological and pathophysiological processes, unraveling of this mechanism leads to a better comprehension of GPCR signaling and might improve the development of therapeutic drugs by limiting side effects [11–14]. For the Y_2_R, a typical pattern has been reported, which involves arrestin-depended internalization after stimulation with its endogenous ligand NPY [19, 35, 36]. Surprisingly, we identified in this study that despite the ligand-dependent internalization, the downstream Gα_i_ pathway remains “turned on”, which is reflected in severely depressed cellular cAMP levels or vice versa elevated inositol phosphate levels when using chimeric Gα_qi_ protein. Moreover, ineffectiveness of adenylyl cyclase activator forskolin to counteract low cAMP levels and re-elevate cellular cAMP amount was observed as well, raising the question of an additional regulatory mechanism.

The concept that GPCRs internalize and continuously activate G proteins was demonstrated first by the experimental data of *Shukla* et al. They confirmed a large complex consisting of G protein, arrestin and receptor simultaneously [37]. Here, the G protein remains bound to the transmembrane core of the receptor while the arrestin interacts with the C-terminal part of the receptor without terminating signaling due to internalization [38]. However, based on this data, *Wanka* et al. recently demonstrated for the Y_2_R that both the receptor C-terminus and the transmembrane core are engaged in arrestin binding [19]. This so-called core-conformation mutually excludes G protein binding to the same receptor.

Nonetheless, our observations of a long-lasting G protein activation, which have been shown for inositol phosphate accumulation as well as for cAMP measurements are in contrast with these findings and doubt the classical role of arrestin-dependent internalization to hamper G protein signaling. Indeed, persistent G protein signaling was already reported for different members of the GPCR-family, e.g. thyrotropin receptor, parathyroid hormone receptors as well as sphingosine 1-phosphate receptor 1, but the prolonged activation was primarily found from intracellular compartments after internalization [39–41]. Since an internalization deficient Y_2_R mutant, lacking the important Ser^374^ and Thr^376/379^ residues within the internalization motif ^373^DSFTEATNV^381^ at the proximal C-terminus [18] displayed a similar activation pattern, we exclude intracellular signaling and rather suggest an additional regulatory mechanism concerning activation and desensitization that is delimited to the cell membrane and primarily independent of arrestin recruitment. Additionally, our hypothesis is strengthened because stimulation of the wild type Y_2_R with very low NPY concentration (10 nM) in a first round already resulted in a prolonged G protein activation. Comparable to the internalization-deficient mutant, the low NPY concentrations are insufficient for the recruitment of arrestin. These results are in accordance with previous investigation of *Walter* et al and *Lundell* et al*,* who postulated an arrestin-dependent internalization only at higher NPY concentration [18, 42]. The low affinity binding of arrestin towards the Y_2_R might provide the basis for a persistent G protein activation as a consequence of delayed arrestin recruitment and hence impaired termination of G protein signaling.

Moreover, another unexpected finding in our study is the non-responsiveness of the adenylyl cyclase that was observed after NPY stimulation of the cells, since the global forskolin-dependent activation of AC was impaired. We speculate that the Gα_i_/Ca^2+^-inhibited AC5/6 subtypes are targeted by Gα_i_ signaling in HEK293 cells, as the cAMP signal was previously shown to be reduced by addition of CaCO_3_, and the cellular cAMP levels are elevated in the presence of a protein kinase C inhibitor, both of which is characteristic for these AC subtypes [43–45] (Figure S5).

Blocking G_i_-signaling by treating the cells with PTX, the adenylyl cyclase was still activatable by forskolin, indicating a receptor mediated regulation. We presume that long-lasting G protein activation of the Y_2_R and therefore the sustained inhibitory effect leads to controlled desensitization of the AC5/6 subtypes preventing overstimulation. However, signaling of co-transfected Gα_s_-coupled MCR1 was only very slightly affected after NPY treatment and stimulation with NAPamide still leads to a robust cAMP response. We suggest that additionally the forskolin-insensitive AC9 subtype contributes to the cAMP signal of this receptor. The AC9 subtype is characterized by its responsiveness to Gα_s_ but not to forskolin [43, 45]. *Mullershausen* et al reported similar findings of AC desensitization of the sphingosine 1-phosphate receptor 1 and postulated that this phenomenon has been detected with several members of G_i_ protein-coupled GPCR and seems to be dependent on the amount of adenylyl cyclase and G_s_ proteins [41, 46, 47]. Moreover *Watts and Neve* et al suggested that this so called heterologous sensitization of adenylyl cyclase following receptor activation is independent of receptor desensitization, internalization and down-regulation and contributes to fundamental physiological processes within the neurotransmitter crosstalk [47]. However, at present, the biological role of Y_2_R-mediated inhibition of the cAMP system remains unknown.

Additionally to this desensitization of the adenylyl cyclase/ cAMP system, we found that after intracellular sorting and subsequent reappearance of the Y_2_R at the cell membrane, ligand binding, G protein activation and receptor internalization was diminished in the second stimulation as well, suggesting that receptor desensitization is regulated independently and controlled by additional factors.

To exclude receptor processing during the endocytosis and recycling route, we used the internalization deficient variant to further characterize desensitization of the receptors. Interestingly, our data clearly demonstrate a significant loss in receptor activation after repeated stimulation, although no endocytosis of the receptors occurred. Moreover, stimulation with 10 nM NPY (insufficient for receptor endocytosis) in the first experiment already resulted in a significantly impaired receptor activation in the followed set-up (Fig. 4c). Based on these findings, we hypothesize an additional desensitization mechanism that controls G protein signaling independently of receptor internalization. Indeed, several studies have already shown desensitization of receptors is not limited to receptor internalization. For different members of the GPCR family like m_2_ muscarinic-, endothelin- as well as adenosine A_2a_ receptors an arrestin- and internalization-independent desensitization mechanism were already described [34, 46, 48, 49]. Moreover, specificity and selectivity of these data were confirmed by co-transfection of Y_1_ and Y_2_ receptors simultaneously. Selective stimulation of Y_2_R with Ahx^5–25^-NPY and subsequent activation of Y_1_R with F^7^P^34^-NPY resulted in a significantly decreased Y_1_ receptor response, whereas vice versa selective first stimulation of Y_1_R and subsequent Y_2_R stimulation did neither affect receptor activation nor AC desensitization, indicating distinct “consumption” of effector proteins within one receptor family. Co-transfection with MCR1- a member of Gα_s_-coupled receptors – and subsequent stimulation of the Y_2_R did not lead to changes in the following cAMP signaling response of MCR1, indicating a mechanism specific for Gα_i_-signaling.

Contrary to the obviously low arrestin binding affinity, the unoccupied receptor apparently exists in a conformation that strongly favors G protein coupling*. Kaiser* et al. recently reported two different affinity states concerning Y_2_R binding properties [29]. Based on our radioligand binding experiments and in agreement with these findings, ^125^I-PYY ligand displacement experiments display a biphasic binding, including a high affinity state of ~ 40% receptors and a low affinity state. Notably, the high affinity state was apparently lost after stimulation with 1 μM NPY, which obviously correlates with the amount of available G protein. High affinity binding is a result of allosteric interactions between the G protein and ligand binding site of the receptor, and thus regulated by the association of G protein. Accordingly, decoupling of G protein-receptor-complex through desensitization is expected to lead to a loss in high affinity binding. Preassembly of the G protein and Y_2_R prior to agonist stimulation and disruption of the high affinity state by GTPγS was already suggested by *Kaiser* et al [29] and is consistent with our findings that demonstrate the shift to low affinity binding sites after first agonist stimulation. Similar results have been obtained for other G_i_-coupled GPCR such as dopamine receptors and opioid receptors, indication a tight correlation between desensitization and changes in agonist-receptor-affinity states [5, 50–53]. Here, we further observed a dramatic decrease in total binding of pretreated receptors (B_max_ 32%). Besides competing with remaining NPY due to inefficient washing, the loss in B_max_ might be attributed to an insufficient ligand affinity in absence of preassembled G protein and would align well with the reported loss in total binding by addition of GTPγS [29]. As receptors that are exposed to agonist for the first time show a high affinity state, we propose that agonist binding results in a strong effector signaling leading to depletion of the intracellular G protein pool. This prevents reassembly of the R-G/ R*-G-complexes after agonist washout, resulting in prolonged desensitization, which is reflected in the loss of the high affinity state. Moreover, this hypothesis is supported by the previously described pattern of prolonged G protein signaling and is further corroborated by the overexpression of chimeric G protein. By varying G protein-receptor ratio towards an oversupply of G protein, we have confirmed a positive correlation with a higher receptor response and an increased E_max_ after pretreatment with 1 μM NPY. Thus, very tight binding of the agonist leads to a long-lasting agonist-receptor-complex, which further activates all the nearby functional G proteins until the G protein pool is depleted. Hence, we postulate a new regulatory mechanism by which the Y_2_R functions as G protein magnet and captures all freely available G proteins. The high intrinsic affinity of the receptor to inhibitory G protein and strong allosteric connections between G protein and the ligand-binding site of the receptor contributes to very efficient activation and turnover of cellular G proteins, resulting in strong and persistent activation of the Gα_i_-pathway. This depletes the functional intracellular G protein repertoire before arrestin-mediated internalization can terminate signaling. Thus, the cell is left in a refractory state, preventing further G_i_-signaling of both the Y_2_R itself but also other Gα_i/o_-coupled receptors, suggesting that Y_2_R expression dominates G_i_-signaling within the cell. Furthermore, our studies highlight that the availability of effector proteins critically affects the cellular signaling status, and simple depletion of a downstream effector adds to the stock of cellular control mechanisms. Up to now, the biological background remains unclear but we postulate an additional controlling mechanism within the presynaptic and postsynaptic transmitter crosstalk as these findings are not only observed in transfected HEK293 cells but also present in SH-SY5Y (bone marrow cells from metastatic neuroblastoma) and SMS-KAN (cells from primary brain tumor), both endogenously expressing the Y_2_ receptor. However, further investigations are necessary to completely unravel the pathway leading to a controlled receptor desensitization versus resensitization.

## Conclusion

Our data demonstrate that activation of the Y_2_R results in a strong and persistent activation of the Gα_i_-pathway. A high intrinsic affinity of the receptor to inhibitory G protein and the strong allosteric interaction between G protein- and the ligand binding site of the receptor contributes to very efficient activation and turnover of cellular G proteins, which furthermore deplete the intracellular G protein repertoire before arrestin-mediated internalization can terminate signaling. Thus, the cell is left in a refractory state, preventing further G_i_-signaling of both the Y_2_R itself but also of other Gα_i/o_-coupled receptors, suggesting that Y_2_R expression dominates G_i_-signaling within the cell. Furthermore, our studies highlight that the availability of effector proteins critically affects the cellular signaling status, and simply depletion of a downstream effector adds to the stock of cellular control mechanism.

## Supplementary information

**Additional file 1: **Supporting Results. **Figure S1.** Internalization behavior of hY_2_ receptor wild type. **Figure S2.** Prolongation of the recovery time is insufficient to entirely regain activatability. **Figure S3.** Y_2_R activation mainly triggers Ca^2+^-influx from extracellular compartments and excludes Ca^2+^ as a limiting factor. **Figure S4.** Activity of selective NPY receptor analogues tested in SHSY5Y cell line endogenously expressing Y_2_R. **Figure S5.** Impact of Ca^2+^ and PKC on cellular cAMP level.

## Data Availability

All other data needed to evaluate the conclusions in the paper are present in the paper or in the supporting Information. This manuscript is accompanied by supporting information including additional pharmacological analysis and characterization of Y_2_R. Even if these results are not essential to understand the manuscript, we think, it is adjuvant to underline the new findings.

## Abbreviations

ACN: Acetonitrile
AC: Adenylyl cyclase
ANOVA: Analysis of variance
Arr: Arrestin
cAMP: Cyclic adenosine monophosphate
CHX: Cycloheximide
DMEM: Dulbecco’s modified Eagle’s medium
eYFP: Enhanced yellow fluorescent protein
Fmoc: Fluorenylmethyloxycarbonyl
Gα_i_: Inhibitory Gα-pathway
Gα_q_: Phospholipase C activating Gα-pathway
GPCR: G protein coupled receptor
GTPγS: Guanosine 5′-O-[gamma-thio] triphosphate
HA: Hemagglutinin
HBSS: Hank’s buffered salt solution
HEK: Human embryonic kidney
IP: Inositol phosphate
LiCl: Lithium chloride
MALDI-ToF: Matrix-assisted laser desorption/time of flight
MCR1: Melanocortin 1 receptor
NH_4_Cl: Ammoniumchloride
NPY: Neuropeptide Y
PLC: Phospholipase C
PP: Pancreatic polypeptide
PTX: Pertussis toxin
PYY: Peptide YY
RP-HPLC: Reversed phase – high performance liquid chromatography
TAMRA: (5,6)-carboxytetramethylrhodamine
YxR: NPY receptor subtype x
w/o: Without
