# Supplementary material for: Unusually persistent Gαi-signaling of the neuropeptide Y2 receptor depletes cellular Gi/o pools and leads to a Gi-refractory state
Source: Cell Commun Signal. 2020 Mar 30;18:49. doi: 10.1186/s12964-020-00537-6 (PMC7104545; doi:10.1186/s12964-020-00537-6)
Supplement: Supplementary file 2 — Additional file 1: Supporting Results. Figure S1. Internalization behavior of hY2 receptor wild type. Figure S2. Prolongation of the recovery time is insufficient to entirely regain activatability. Figure S3. Y2R activation mainly triggers Ca2+-influx from extracellular compartments and excludes Ca2+ as a limiting factor. Figure S4. Activity of selective NPY receptor analogues tested in SHSY5Y cell line endogenously expressing Y2R. Figure S5. Impact of Ca2+ and PKC on cellular cAMP level. [file 12964_2020_537_MOESM2_ESM.docx]

**Additional file 1**

Supplementary Materials:


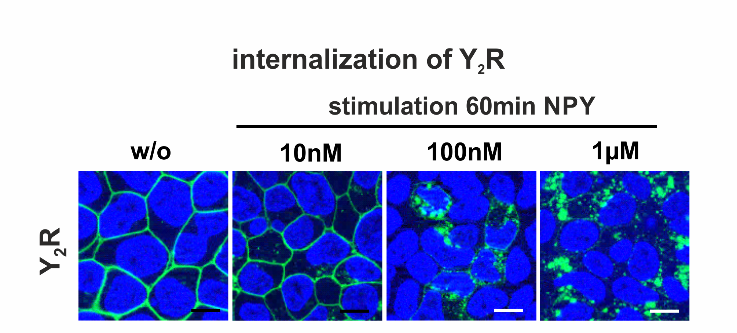


**Fig. S1. Internalization behavior of hY_2_ receptor wild type.** Receptor internalization was analyzed in transiently transfected HEK293 cells by fluorescence microscopy prior (w/o) and after 60 min stimulation with different NPY concentrations. Experiments represent data n>2 (scale bar: 10µm).


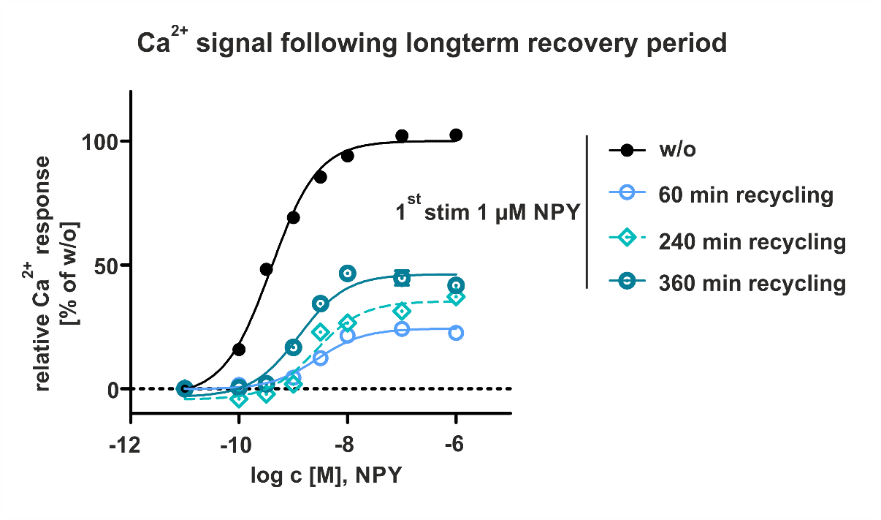


**Fig. S2. Prolongation of the recovery time is insufficient to entirely regain activatability.** Concentration-response curves of transiently co-transfected HEK293-hY_2_R + Gαqi were measured by Ca^2+^-flux assay using the chimeric G protein (GαΔ6qi4myr) as a tool. Decreased Ca^2+^ response from transiently transfected HEK293-hY_2_R-eYFP cells was obtained after stimulation with 1 µM (light blue) for 60 min at 37 ° C followed by subsequent washing and 60 min recycling. Elongation of the period of recovery led to a partial gain of Ca^2+^ signal after stimulation with 1 µM NPY, followed by washing steps but did not exceed 50 % of control cells without NPY stimulation (black). Experiments represent data n≥3.


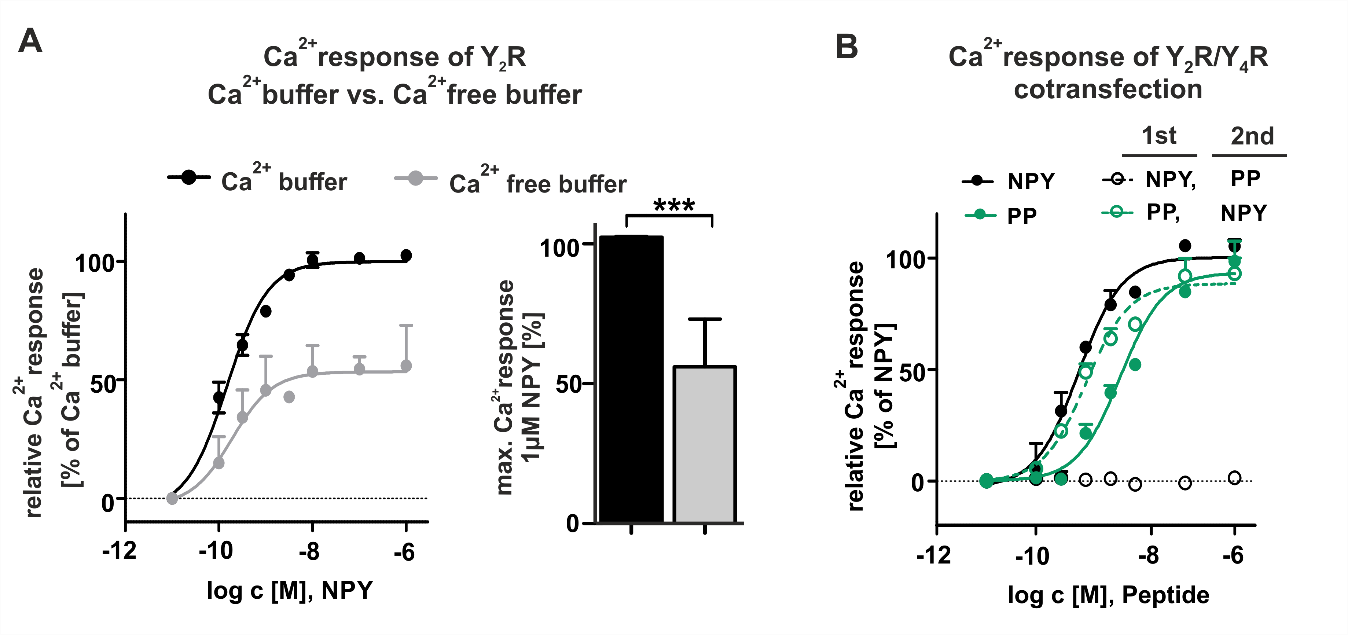


**Fig. S3. Y_2_R activation mainly triggers Ca^2+^-influx from extracellular compartments and excludes Ca^2+^ as a limiting factor.** Decreased Ca^2+^ response from transiently transfected HEK293-hY_2_R-eYFP cells was obtained after stimulation of cells with NPY using Ca^2+^-free buffer. Ca^2+^activation was determined by Ca^2+^-flux assay and maximum Ca^2+^response was calculated by saturation curves measured at a concentration of 1µM NPY. B) Concentration-response curves of transiently co-transfected HEK293-hY_2_R/hY_4_R were measured by Ca^2+^ flux assay. Cells without first stimulation ( NPY; black line; PP, green line) or with first stimulation with 1 µM PP, the endogenous selective Y_4_R peptide, subsequent acidic wash and 60 min recovery period, followed by stimulation with NPY to address Y_2_R (dashed green line) exhibit no significant activity loss. However, first stimulation with 1 µM NPY, subsequent washing and 60 min incubation in ligand free media, followed by stimulation with PP (open black circle) revealed an obvious loss in activity, since no Ca^2+^response was measured. Experiments represent data n≥2; significance was determined by one-way ANOVA, Tukey post test, ***: P<0.0001.

**
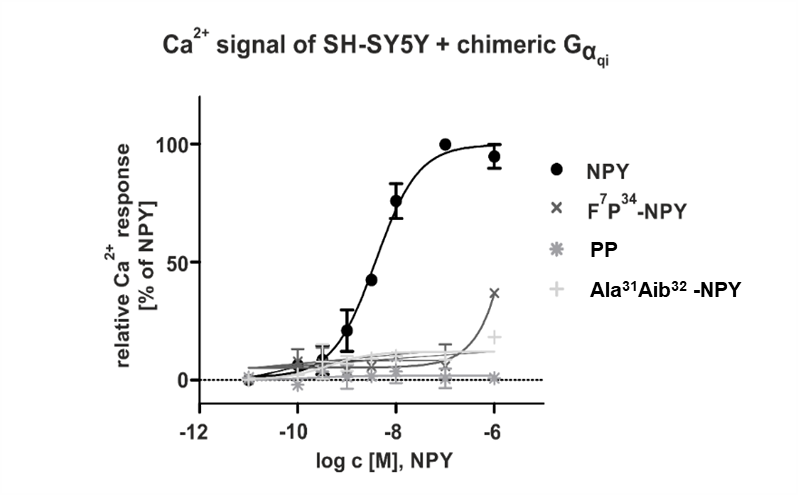
**

**Fig. S4. Activity of selective NPY receptor analogues tested in SHSY5Y cell line endogenously expressing Y_2_R.** Concentration-response curves of transiently co-transfected SHSY5Y cells with the chimeric G protein (GαΔ6qi4myr) as a tool were measured by Ca^2+^-flux assay. Specific Y_2_R signaling was investigated by using different receptor subtype selective NPY ligands. Treatment with either [F^7^,P^34^]-NPY, a selective Y_1_R agonist (dark grey) or PP (grey), the endogenous ligand of Y_4_R, or [Ala^31^, Aib^32^]‐NPY (light grey), which prefers Y_5_ receptor proved Y_2_R selectivity. Experiments represent data n≥3.


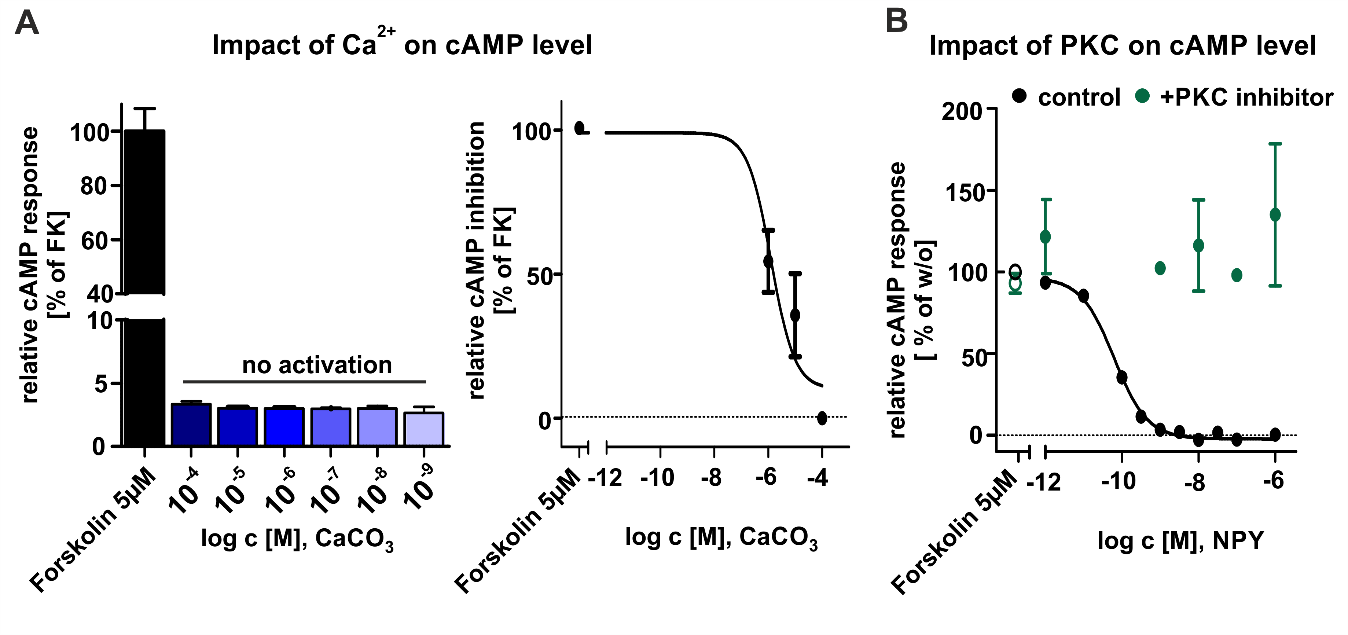


**Fig. S5. Impact of Ca^2+^ and PKC on cellular cAMP level.** A) Adenylyl cyclase activity in transiently transfected HEK293-hY_2_R-eYFP cells was measured with different concentration of CaCO_3._ No stimulatory activation of adenylyl cyclase was observed by incubation cells with concentration of 1 nM up to 100 µM CaCO_3_ (blue) compared to 5 µM FK (100%, black bar, left panel). Adenylyl cyclase inhibition was measured by stimulating cells with 5 µM FK first and subsequent treatment with concentration of 1 µM up to 100 µM CaCO_3_ (middle panel). B) The influences of PKC was investigated by using Bisindolylmaleimide I as PKC inhibitor. Transiently transfected HEK293-hY_2_R-eYFP were incubated with 10 µM PKC inhibitor 30min prior stimulation with peptide. Using PKC inhibitor abolished inhibitory effect of adenylyl cyclases and Y_2_R downstream signaling. Experiments represent data n≥2.
